# Tourniquet-Applied Upper Limb Orthopaedic Surgery Results in Increased Inflammation and Changes to Leukocyte, Coagulation and Endothelial Markers

**DOI:** 10.1371/journal.pone.0011846

**Published:** 2010-07-28

**Authors:** Stephen F. Hughes, Beverly D. Hendricks, David R. Edwards, Jim F. Middleton

**Affiliations:** 1 Department of Biological Sciences, University of Chester, Chester, United Kingdom; 2 Haematology Department, Glan Clwyd Hospital, Conwy, United Kingdom; 3 Haematology Department, Gwynedd Hospital, Gwynedd, United Kingdom; 4 Leopold Muller Arthritis Research Centre, The Robert Jones and Agnes Hunt Orthopaedic Hospital, Medical School, Keele University, Staffordshire, United Kingdom; Emory University, United States of America

## Abstract

**Purpose:**

During this pilot clinical study, patients scheduled for elective tourniquet-applied upper limb orthopaedic surgery were recruited to investigate the effects of surgery on various biological markers (n = 10 patients).

**Methods:**

Three venous blood samples were collected from the arm at the ante-cubital fossa, at baseline (pre-operatively), 5 and 15 minutes after reperfusion (post-operatively). Neutrophil and monocyte leukocyte sub-populations were isolated by density gradient centrifugation techniques. Leukocyte activation was investigated by measuring the cell surface expression of CD62L (L-selectin), CD11b (Mac-1) and the intracellular production of hydrogen peroxide (H2O2), via flow cytometry. C-reactive protein (CRP) was measured using a clinical chemistry analyser. Plasma concentrations of protein C and von Willebrand factor (vWF) were measured using enzyme-linked fluorescent assays (ELFA).

**Results:**

Following tourniquet-applied upper limb orthopaedic surgery, there was a decrease in neutrophil CD62L expression (p = 0.001), an increase in CD11b expression and in the intracellular production of H2O2 by neutrophils and monocytes (p<0.05). An increase in CRP concentration (p<0.001), a decrease in protein C concentration (p = 0.004), with a trend towards elevated vWF levels (p = 0.232) were also observed during this time.

**Conclusions:**

Conventionally, patients undergoing orthopaedic surgery have been monitored in the peri-operative period by means of CRP, which is a non-specific marker of inflammation. This test cannot differentiate between inflammation due to current or pre-existing disease processes and the development of ischaemia-reperfusion injury surgery. The findings from this study suggest that markers such as CD11b, protein C and H_2_O_2_ may provide alternative ways of assessing leukocyte and coagulation activation peri-operatively. It is proposed that by allowing orthopaedic surgeons access to laboratory markers such as CD11b, protein C and H_2_O_2_, an accurate assessment of the extent of inflammation due to surgery *per se* could be made.

## Introduction

With respect to tourniquet-applied orthopaedic surgery, comparing a range of biological markers as part of the post-operative period has not yet been extensively researched. CD62L, CD11b and the intracellular production of H_2_O_2_ were measured to assess leukocyte function. CRP was measured as a marker of non-specific inflammation, with Protein C and VWF assessing coagulation and endothelial activation respectively. Measurement of various parameters following upper limb surgery may therefore provide a useful tool, as indicative markers following tourniquet-applied orthopaedic surgery.

Neutrophils and monocytes are components of the non-specific immune system and are capable of phagocytosis. Both neutrophils and monocytes have been implicated to play a key role in the development of the inflammatory response post surgery, where they are intrinsically involved in leukocyte-endothelial cell interactions [1]–[4]. During an inflammatory response it can be appreciated that interactions between the phagocytic leukocyte and the endothelium involve the expression of various adhesion molecules. Specific adhesion molecules important in mediating adhesive interactions include CD62L (L-selectin) and CD11b (Mac-1) on neutrophils and monocytes, which bind to their corresponding counter-receptors to facilitate leukocyte-endothelial cell interactions [5]–[8]. The adhesion of leukocytes to the endothelium is associated with neutrophil and monocyte activation, which leads to the respiratory burst and subsequent production and release of reactive oxygen intermediates (ROIs), such as hydrogen peroxide and superoxide [9]–[11].

Measurement of various biological markers such as CRP, protein C and vWF may provide important information for assessing the inflammatory response post tourniquet-applied upper limb orthopaedic surgery. CRP is produced in the liver and is a member of the class of acute phase reactants. CRP is used mainly as a marker of non-specific inflammation and measuring its values can prove useful in determining disease progress or the effectiveness of treatments. Protein C is a glycoprotein found in plasma, which is synthezised by hepatocytes in the liver. Protein C is a major physiological anticoagulant. It is a vitamin K- dependent serine protease enzyme that is activated at the vascular endothelium by its natural activator, thrombin, into activated protein C (APC). The activated form degrades factor Va and factor VIIIa and prevents blood clots during the coagulation cascade. APC provides physiologic anti-thrombotic activity and exhibits anti-inflammatory and anti-apoptotic activities [12]–[13]. von Willebrand Factor (vWF) is a large multimeric glycoprotein and performs essential functions of haemostasis *in vivo*, through its variety of bridging and binding activities. Specific functions of vWF include its ability to act as an essential co-factor for factor VIII in the blood coagulation cascade, platelet adhesion, platelet aggregation and platelet plug stabilisation. vWF has been reported as being an established marker of endothelial activation [2], [13]–[15].

Tourniquet-applied upper limb orthopaedic surgery is performed to allow surgeons with a bloodless field to perform their work. During tourniquet-applied surgery it can be appreciated that ischaemia-reperfusion injury is an underlying process that also occurs. Ischaemia is the reduction of blood supply to a part of the body and reperfusion occurs when blood flow is re-established to ischaemic tissue. As a result of ischaemia and reperfusion an inflammatory reaction frequently occurs, and it is during reperfusion that increased leukocyte and endothelial activation is understood to occur [2], [16]–[21].

A previous study demonstrated that following a mild ischaemic insult (employing a non-surgical model), the leukocyte response was immediate followed by evidence of leukocyte activation and changes in inflammatory and coagulation markers [21]. The present report builds on previous work and consists of a human study that employed an adapted model of ischaemia-reperfusion injury in a clinical setting, and by measuring additional biological parameters to accurately assess the acute phase response post surgery. The main aim of this pilot clinical study was to test the hypothesis that tourniquet-applied upper limb orthopaedic surgery results in an acute inflammatory response.

## Methods

### Subject Volunteers

Written ethical approval for this pilot analysis study was received from the local research ethics committee (North Wales Central Research Ethics Committee, Reference Number - 05/WNo02/26). Ten volunteers scheduled for elective tourniquet-applied upper limb orthopaedic surgery were recruited after obtaining written informed consent. 5 patients (3 males and 2 females) were scheduled for carpal tunnel decompression surgery and 5 patients (3 males and 2 females) for fasciectomy surgical procedures. The test subjects were aged between 31 and 85 years old (mean age = 58) and all were non-smokers. None had any history of cardiovascular disease.

### Blood collection and cell counting

Venous blood samples were collected into vacutainers containing di-potassium ethylene diamine tetra-acetic acid (EDTA) (1.5mg/ml), tri-sodium citrate (0.11mol/l) and serum clot activator (Greiner Bio-one, UK). Full blood counts were performed using a Coulter® MicoDiff^18^ automated cell counter (Beckman Coulter, UK).

### Tourniquet-applied upper limb orthopaedic surgery

Prior to tourniquet-applied upper limb orthopaedic surgery an 18GA cannula (BD VenflonTM, Sweden) was inserted into the surgical arm at the ante-cubital fossa. A venous blood sample was then collected, which stood as a baseline (pre-operative) measurement for that particular patient. In theatre, a tourniquet was set around the upper arm using nerve block anaesthesia. The tourniquet was set and inflated to 250 mmHg, to ensure a bloodless field prior to surgery. The mean time of ischaemia was 32.30±4.16 minutes (n = 10 patients). Upon the release of the tourniquet, further blood samples were collected by means of the cannula, at 5 and 15 minutes reperfusion (post-operatively). The baseline (pre-operative) samples served as the control measures during this study, with the main aim being to compare the effects of tourniquet-applied surgery on the biological parameters measured within each subject pre and post-operatively.

### Preparation of cell suspensions

Purified neutrophils and mononuclear cell suspensions were prepared by density gradient sedimentation on ficoll hypaque solutions as described by Lennie *et al*, (1987) [22]. Following isolation, cells were re-suspended in phosphate buffered saline (PBS) supplemented with di-potassium EDTA (1.5mg/ml) to yield a final cell count of 2×10^6^ cells/ml. All chemicals were supplied by Sigma-Aldrich, UK. Whole blood samples were separated directly from the fresh samples collected.

### Measurement of cell surface expression of CD62L and CD11b

The monoclonal antibodies used were mouse anti-human CD62L (MCA1076F) and CD11b (MCA551F). The isotype-matched controls used were IgG2b (MCA691F-CD62L) and IgG1 (MCA928F-CD11b). All reagents were purified immunoglobulin/fluorescein isothiocyanate (Ig/FITC) conjugates (AbD Serotec Ltd., U.K.). Isolated leukocyte subpopulations (neutrophils and monocytes) were incubated in monoclonal antibodies at 0.1mg/ml for 30 minutes at room temperature, prior to assay analysis using flow cytometry of gated monocytes and neutrophils as previously defined [2], [21]. Changes in the cell surface expression of CD62L and CD11b (represented by mean fluorescent intensity) were measured at each time interval to assess the effect of surgery on these adhesion molecules.

### Measurement of intracellular hydrogen peroxide production (H_2_O_2_)

Cells were isolated and intracellular H_2_O_2_ production was assessed by adaptation of a technique previously described by Bass *et al* (1983) [23]. The assay was based on the oxidation of non-fluorescent 2′, 7′-dichlorofluoroscin diacetate (DCFH-DA) by H_2_O_2_ to stable and fluorescent dichlorofluoroescein. H_2_O_2_ production was assessed in cells using a fixed volume of 0.5 ml cell suspension (2×10^6^ cells/ml) mixed with 0.5 ml DCFH-DA (20µM) in PBS. Cells were incubated in the dark, at 37°C for 30 minutes before immediate measurement using flow cytometry of gated monocytes and neutrophils.

### Measurement of C-reactive protein (CRP)

Measurement of C-reactive protein was performed using an ILAB 600 clinical chemistry analyser (Instrumentation Laboratory, UK). Highly sensitive CRP was measured using the Quantex CRP plus kits (Reference number: 3000-2209), which were supplied by Bio-kit (Spain) and involved using a turbidimetric assay as previously described by Price *et al* (1987) [24].

### Measurement of plasma concentrations of protein C and von Willebrand Factor (vWF)

Blood samples were collected into tri-sodium citrate or EDTA tubes and were centrifuged separately at 1500*g* for 10 minutes within 4 hours of blood collection. Plasma was removed and stored at −30°C. Plasma concentrations of protein C and vWF were measured by a two step enzyme immunoassay sandwich method, with a final fluorescent detection as described by others [12]–[13]. Measurement of these parameters was performed using a Mini-Vidas automated immunoassay system that uses ELFA (Enzyme-Linked Fluorescent Assay) technology. The Mini-Vidas system and immunoassay kits were supplied from Biomerieux, UK.

### Statistical analysis

Subject volunteers were recruited at random without consideration to gender and age, to reflect the diversity of the demographics of the patients scheduled for elective tourniquet-applied upper-limb orthopaedic surgery. A limiting factor of this study was the relatively small number of patients recruited (n = 10). It would also have been interesting to have followed up the patients with regards to measurement of their biological markers at review clinic's, this could have indicated any continued inflammatory reactions post surgery, which may have had an impact in supporting surgeons with their management strategies of patients during the post-operative period.

During this study, all results were presented as mean ± standard deviation (SD). Initially all the data were tested to determine if the results were normally distributed. The Shapiro-Wilk statistical test was employed with data being considered as normally distributed if p≥0.05. Where data were normally distributed, repeated measures one-way analysis of variance (ANOVA) between samples test was employed adopting a 5% level of significance. Post hoc testing was conducted using the Tukey test for pairwise comparisons between means. During analysis, patient data were fitted as a random variable with the time as the fixed variable.

Data that did not comply with normality were analysed using the Friedman test. Where the Friedman test resulted in statistical significance, subsequent tests were performed using the Wilcoxon test. Statistical significance was accepted when p≤0.05.

## Results

### Effect of tourniquet-applied upper limb orthopaedic surgery on CD62L (L-selectin) and CD11b (Mac-1) cell surface expression of neutrophils and monocytes

**Figure 1:**
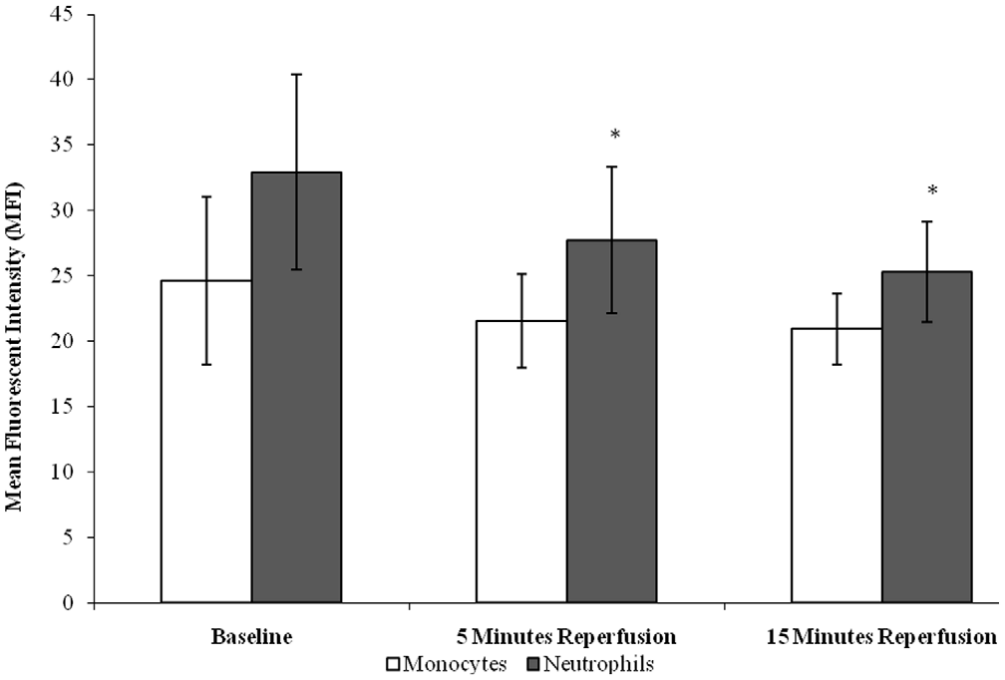
Effect of tourniquet-applied upper limb orthopaedic surgery on CD62L cell surface expression of neutrophils and monocytes. The results are expressed as mean fluorescent intensity (MFI). The points represent mean ± SD, p = 0.001 (neutrophils) and p = 0.061 (monocytes), as determined by ANOVA.

Following tourniquet-applied upper limb orthopaedic surgery CD62L expression decreased on neutrophils (p = 0.001) with a trend in expression being observed in monocytes (p = 0.061), from baseline (pre-operative) (32.94±7.49 neutrophils; 24.64±6.46 monocytes), during 5 minutes (27.72±5.59 neutrophils; 21.55±3.57 monocytes) and 15 minutes (25.35±3.84 neutrophils; 20.94±2.71 monocytes) reperfusion (post-operative), (Figure 1). Upon further testing by pairwise comparisons, there were changes between neutrophil baseline *vs* 5 minutes reperfusion (p = 0.024) and 15 minutes reperfusion (p = 0.003). CD62L cell surface expression in neutrophils appeared to demonstrate higher expression in comparison to monocytes.

**Figure 2:**
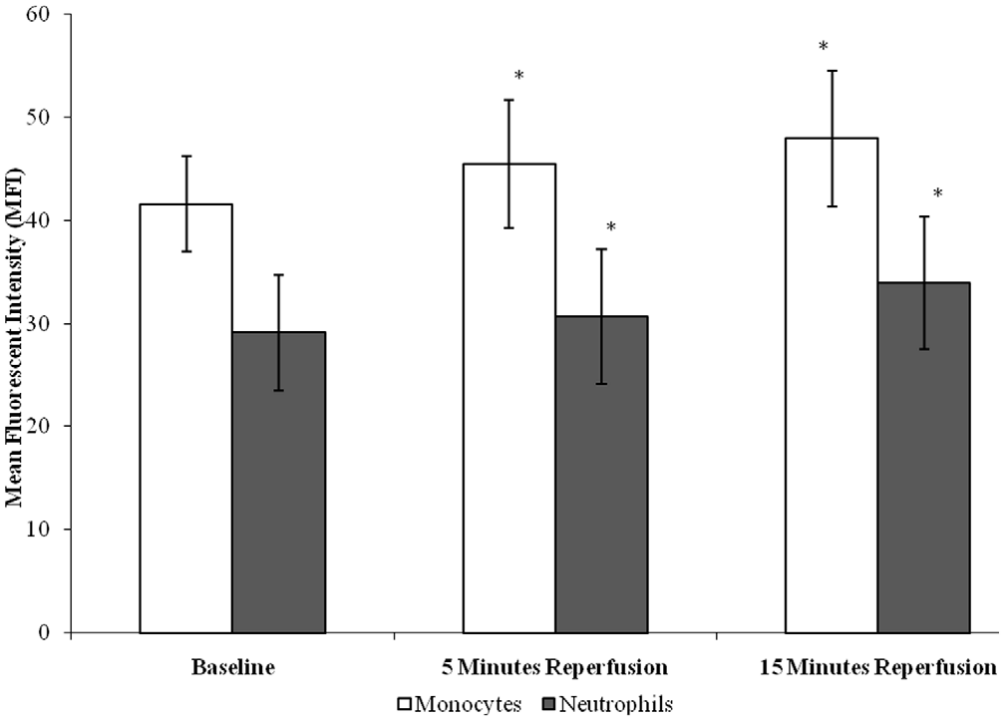
Effect of tourniquet-applied upper limb orthopaedic surgery on CD11b cell surface expression of neutrophils and monocytes. The results are expressed as mean fluorescent intensity (MFI). The points represent mean ± SD, p<0.05 for both neutrophils and monocytes as determined by ANOVA.

There was an effect of tourniquet-applied upper limb orthopaedic surgery on CD11b cell surface expression of both neutrophils and monocytes (p<0.05), (Figure 2). Results demonstrate that neutrophil CD11b expression increased from baseline (29.11±5.61), during 5 minutes (30.71±6.54) and 15 minutes (33.95±6.44) reperfusion, whilst monocyte CD11b expression increased from baseline (41.61±4.61), during 5 minutes (45.46±6.23) and 15 minutes (47.97±6.60) reperfusion. Upon further testing by pairwise comparisons, there were changes between baseline *vs* 5 minutes (p = 0.042 neutrophils; p = 0.009 monocytes) and 15 minutes (p = 0.001 neutrophils; p = 0.002 monocytes) reperfusion for both neutrophils and monocytes. The CD11b cell surface expression in monocytes was consistently higher than that seen in neutrophils, which may be due to the fact that monocytes are larger than neutrophils and may express more CD11b on their surfaces [2], [21].

### Effect of tourniquet-applied upper limb orthopaedic surgery on leukocyte H_2_O_2_ production

**Figure 3:**
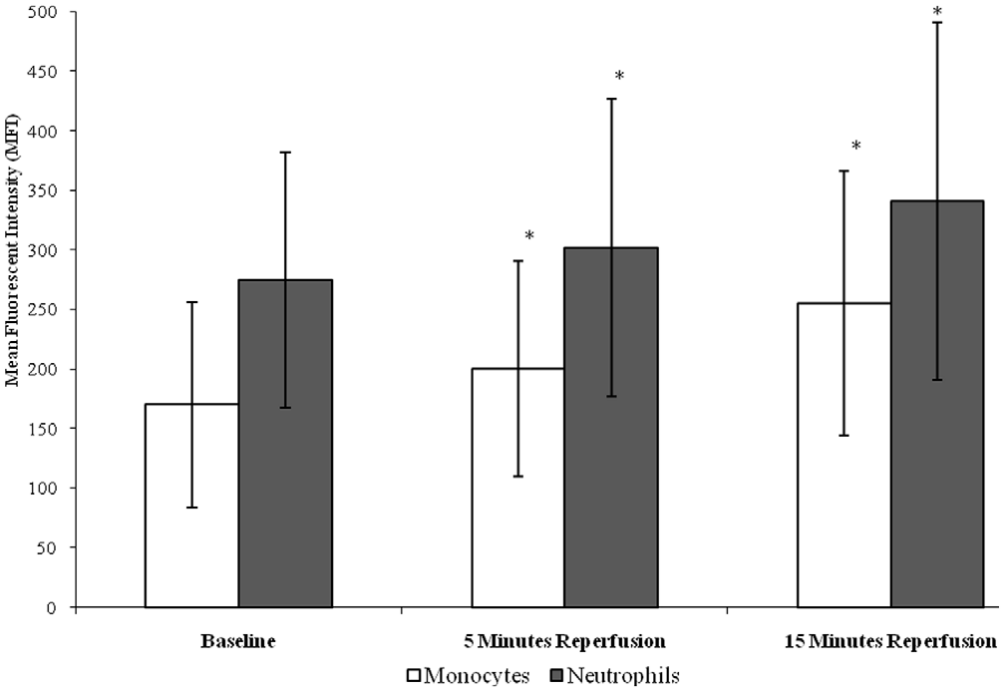
Effect of tourniquet-applied upper limb orthopaedic surgery on intracellular H_2_O_2_ production of neutrophils and monocytes. The results are expressed as mean fluorescent intensity (MFI). The points represent mean ± SD, p<0.001 for both neutrophils and monocytes, as determined by ANOVA and the Friedman test respectively.

Results demonstrate that following tourniquet-applied upper limb orthopaedic surgery there was an increase in the intracellular H_2_O_2_ production of both neutrophils and monocytes (p<0.001), (Figure 3). Specifically, neutrophil intracellular H_2_O_2_ production increased from baseline (274.76±107.14), during 5 minutes (301.58±125.09) and 15 minutes (340.54±150.76) reperfusion, whilst monocyte intracellular H_2_O_2_ production increased from baseline (170.33±86.22), during 5 minutes (200.09±90.95) and 15 minutes (255.21±111.97) reperfusion.

Upon further testing by pairwise comparisons, there were changes between baseline *vs* 5 minutes (p = 0.036) and 15 minutes (p = 0.007) reperfusion for neutrophils. Statistical testing using the Wilcoxon test displayed changes between baseline *vs* 5 minutes (p = 0.005) and 15 minutes (p = 0.005) reperfusion for monocytes.

Interestingly, the intracellular H_2_O_2_ production in neutrophils was consistently higher than that seen in monocytes, which may be due to neutrophils being considered ‘in general’ to be more competent in producing potent biologically active agents such as H_2_O_2_ [2], [21].

### Effect of tourniquet-applied upper limb orthopaedic surgery on the inflammatory response, coagulation activity and endothelial activation

**Figure 4:**
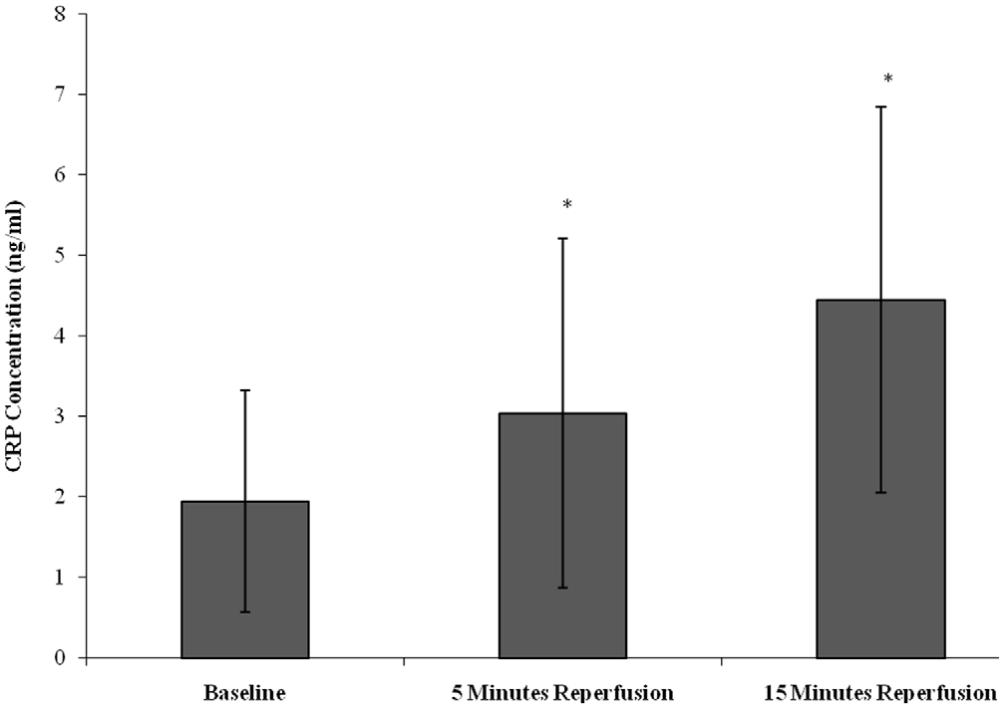
Effect of tourniquet-applied upper limb orthopaedic surgery on C-reactive protein (CRP) concentration. The results are expressed as CRP concentration (ng/ml). The points represent mean ± SD, p<0.001, as determined by the Friedman test.

CRP was measured as marker of non-specific inflammation (Figure 4). CRP concentration increased from baseline (1.95±1.38), during 5 minutes reperfusion (3.04±2.17) and 15 minutes reperfusion (4.45±2.45). Statistical significance was achieved (p<0.001), as determined by the Friedman test. Upon further testing the Wilcoxon test showed differences between baseline *vs* 5 minutes (p = 0.015) and 15 minutes (p = 0.005) reperfusion.

**Figure 5:**
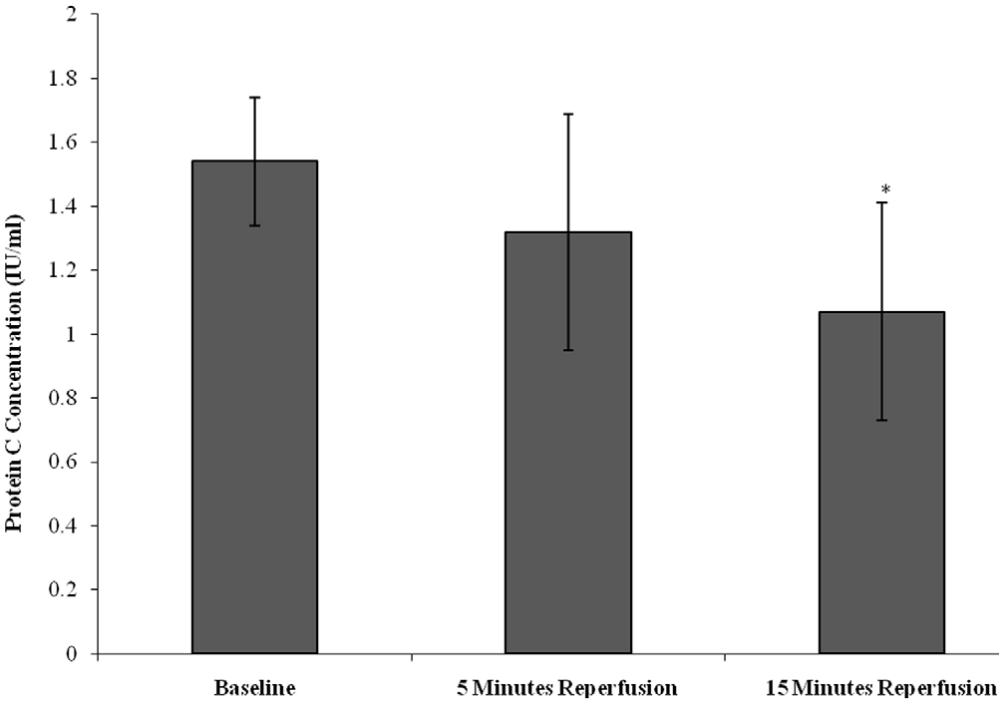
Effect of tourniquet-applied upper limb orthopaedic surgery on protein C concentration. The results are expressed as protein C concentration (IU/ml). The points represent mean ± SD, p = 0.004, as determined by ANOVA.

Protein C was measured as marker of coagulation activity (Figure 5). Protein C concentration decreased from baseline (1.54±0.25), during 5 minutes reperfusion (1.32±0.37) and 15 minutes reperfusion (1.07±0.34). Statistical significance was achieved (p = 0.004), as determined by ANOVA. Upon further testing pairwise comparison testing showed differences between baseline *vs* 15 minutes reperfusion (p = 0.011).

**Figure 6:**
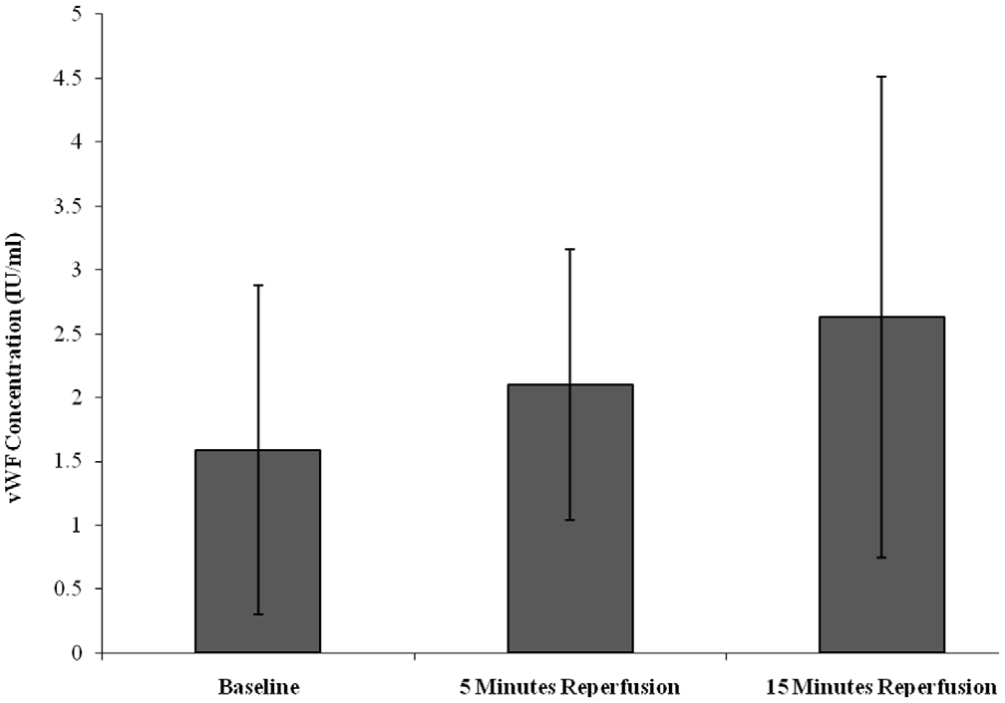
Effect of tourniquet-applied upper limb orthopaedic surgery on vWF concentration. The results are expressed as vWF concentration (IU/ml). The points represent mean ± SD, p = 0.232, as determined by the Friedman test.

Endothelial activation following tourniquet-induced upper limb orthopaedic surgery was assessed via measurement of vWF concentration, which is an established marker of endothelial activation (Figure 6) [2], [14], [21]. vWF levels increased from baseline (1.58±1.29), during 5 minutes (2.10±1.06) and 15 minutes (2.63±1.88) reperfusion. Although none of these changes reached statistical differences (p = 0.232), a trend of increasing vWF concentration was observed from baseline and 15 minutes reperfusion.

## Discussion

An important aspect of this study was to provide a better understanding of the inflammatory response following tourniquet-applied upper limb orthopaedic surgery. Using patients scheduled for elective surgery, we established that neutrophils and monocytes analysed *ex vivo* showed evidence of changes in markers associated with adhesion and activation. These were linked with changes in markers of inflammatory response and coagulation activity, and there was a trend towards endothelial activation.

It can be appreciated that the adhesion and transendothelial migration of leukocytes into the surrounding tissues are crucial steps in inflammation and have also been reported to play a key role during episodes of ischaemia-reperfusion injury [16]–[21]. The present study was designed to ascertain whether tourniquet-applied upper limb orthopaedic surgery results in changes in the cell surface expression of the CD62L and CD11b adhesion molecules post surgery. During this investigation there was evidence of decreased CD62L expression at the cell surface of neutrophils probably due to shedding of this adhesion molecule. However, another explanation of the decrease observed in the CD62L cell surface expression of neutrophils could be that when endothelial cells become activated, the subset of neutrophils with the highest expression of CD62L may tend to roll and adhere which would leave the cells with lesser CD62L-expression in the circulation available for blood sampling. Our results support the evidence that CD62L plays a key role during the early stages of the leukocyte adhesion cascade, which facilitates leukocyte adhesion to the endothelium, and provides further evidence of an involvement between these cells during an inflammatory response post surgery [21]. Our results are in accord with Seekamp *et al* [25] who showed a reduction, although not significant, in CD62L expression on neutrophils following elective knee surgery without the application of a tourniquet. Our data show a more significant reduction of CD62L on neutrophils and monocytes following upper limb surgery which involved the use of a tourniquet.

Results from the present study demonstrate an increased expression of the adhesion molecule CD11b by both neutrophils and monocytes following tourniquet-applied upper limb orthopaedic surgery. This adhesion molecule on the cell surface of phagocytic leukocytes may bind to counter-receptors, such as intercellular adhesion molecule-1 (ICAM-1), present on the surface of vascular endothelium. This interaction would mediate firm adhesion of the leukocyte to the endothelium, aiding the inflammatory response post surgery. Thus increased expression of CD11b and decreased expression of CD62L by neutrophils and monocytes may play a central role as the mechanism by which leukocyte adhesion and consequently activation occurs following surgery [2], [21], [26]. Our results are in line with those of Sutter *et al* [26] who found increased cell surface expression of CD11b and CD18 by granulocytes and monocytes after tourniquet ischaemia during elective hand surgery.

Changes in the cell surface expression of CD62L and CD11b following tourniquet-applied upper limb orthopaedic surgery is associated with cell activation. This was indicated by the increase in intracellular H_2_O_2_ production of monocytes and neutrophils. In addition, during leukocyte activation it can be appreciated that further bioactive material, such as other ROIs and proteolytic enzymes are released extracellularly [2], [10], [15], [21], [23]. Collectively, the actions of these degradative substances may potentially cause considerable damage to host tissue and prolong the inflammatory response following surgery. Measurement of the intracellular production of H_2_O_2_ by phagocytic leukocytes may therefore provide a useful marker that could be applied to monitor the acute inflammatory response following tourniquet-induced surgery of the upper limbs [21].

In support of the investigations to assess leukocyte function following surgery the total leukocyte concentration in blood decreased from baseline to 5 minutes reperfusion (data not shown). Evidence of changes to the leukocyte concentration was also supported by the reduction in granulocyte, lymphocyte and monocyte individual cell counts. However, these changes in total and individual leukocyte counts were slight and not significant. These trends agree with the above changes in CD11b, which may reflect an increase in rolling and adhesion as those cells would not have been found in the blood samples collected.

Another aspect of this study was to assess markers of the inflammatory response and coagulation activity, and any endothelial activation that may be incurred. Conventionally, patients undergoing orthopaedic surgery have been monitored in the peri-operative period by means of CRP, which is a non-specific marker of inflammation. A previous study has been performed to measure post operative plasma CRP levels following various elective orthopaedic surgical procedures [27]. During this study plasma CRP levels were measured prospectively at the preoperative day and at the 1^st^, 2^nd^, 3^rd^, 7^th^ and 15^th^ postoperative days. Results demonstrated that plasma CRP levels fluctuated , with maximum values observed between the 2^nd^ and 3^rd^ postoperative day, followed by a steady decrease. Changes to CRP concentration reported during the present study demonstrate that there was an increased inflammatory response post surgery, although clinically these may not be of significant concerns as all results are within the normal clinical reference range. The reason for the CRP levels being within the normal range may be the early time points sampled (up to 15 minutes post-surgery) since maximum levels of CRP may be developed later at 2–3 days [27]. The elevated levels of CRP observed may be due to the release of pro-inflammatory cytokines such as interleukin-1 and -6 (IL-1, IL-6) from the activated cells in response to surgery and inflammation [21], [25].

There is a lack of studies on the effects of tourniquet-applied upper limb surgery on circulating protein C levels. This protein can be regarded as an important physiological anticoagulant, and a decrease in the concentration demonstrated during the present study suggests an increase in coagulation activation following surgery. At this time it can be appreciated that individuals may be subjected to increased bleeding tendencies during the peri-operative period. Evidence of endothelial activation following tourniquet-induced ischaemia- reperfusion injury has previously been documented using a non-surgical human model [2], [21]. The results from our study complement these studies and provide further evidence of a trend toward endothelial activation, which was demonstrated by an increase in vWF levels following tourniquet-applied upper limb orthopaedic surgery.

One of the limiting factors of this study was that reperfusion samples were collected during only short periods upon the release of tourniquet following surgery (i.e. 15 minutes). It would have been interesting to have followed up the patients with regards to measurement of their biological markers at review clinics, this could have indicated any continued inflammatory reactions post surgery, which may have had an impact in supporting surgeons with their management strategies of patients during the post-operative period.

However, it is proposed that changes in the measured parameters during this study may not be due to a single factor, but due to a number of factors that may result following surgery such as: tissue damage, wound repair and ischaemia-reperfusion injury experienced during upper limb orthopaedic surgery.

In conclusion, this study demonstrates that following tourniquet-applied upper limb orthopaedic surgery, neutrophils and monocytes are rapidly activated and produce potent reactive oxygen intermediates. This was associated with evidence of increased inflammation and coagulation activity, with a trend toward endothelial activation. We report that biological markers such as CD11b, protein C and H_2_O_2_ may provide alternative ways of assessing leukocyte and coagulation activation post-operatively. It is proposed that by allowing orthopaedic surgeons access to these laboratory markers, an accurate assessment of the extent of inflammation following surgery *per se* may be made. The biological markers assessed during this investigation may have a role in monitoring potential infectious complications that can occur during the postoperative period.
